# Supplementary material for: Insights into the Synergistic Antibacterial Activity of Silver Nitrate with Potassium Tellurite against Pseudomonas aeruginosa
Source: Microbiol Spectr. 2023 Jul 6;11(4):e00628-23. doi: 10.1128/spectrum.00628-23 (PMC10433965; doi:10.1128/spectrum.00628-23)
Supplement: Supplemental file 1 — Supplemental material. Download spectrum.00628-23-s0001.docx, DOCX file, 4.4 MB [file spectrum.00628-23-s0001.docx]

***Supplementary material for***

**Insights into the synergistic antibacterial** **activity of silver nitrate with potassium tellurite against *Pseudomonas aeruginosa***

Ali Pormohammad, Andrea Firrincieli, Daniel A. Salazar-Alemán, Mehdi Mohammadi, Dave Hansen, Martina Cappelletti, Davide Zannoni, Mohammad Zarei, Raymond J. Turner

**Supplementary materials and methods**

***Bacterial strain, culture media, stock and working metal (loid)-based antibiotics (MBAs)*** ***solutions.***

Bacterial strains were stored at − 70 °C in Micro-bank vials as described by the manufacturer (proLab Diagnostics, Richmond Hill, ON, Canada). *P. aeruginosa* ATCC 27853, PAO1, and 39 clinical isolates were used in this study. Simulated wound fluid (SWF) [50% peptone water (0.85% NaCl, 0.5 g peptone per 500.0 mL):50% foetal calf serum (GIBCO, Thermo Fisher Scientific, Waltham, MA, USA, Lot# 2212202RP)] was used as the growth medium and susceptibility testing media in this study (1, 2). Two metal(loid)-based antimicrobials (MBAs) are used in this study. Silver nitrate (AgNO_3_; abbreviated to **Ag**, Sigma-Aldrich, St Louis, MO, USA Lot# 39F-3539), and potassium Tellurite (K_2_TeO_3_; **Te**), Sigma-Aldrich, St Louis, MO, USA, Cat# P0677-25G). Where relevant, two antibiotics were used as comparators: gentamicin (gen) and ciprofloxacin (cip). Stock solutions were made up to 100 mM in distilled and deionized (dd) H_2_O; working solutions were made up to 10 mM in SWF. All stock metal(loid) dilutions were stored in glass vials stored at room temperature and dark place for no longer than 2 weeks. No more than 30 min before experimental use, working solutions were made from stock metal(loid) solutions.

***Minimum Inhibitory Concentration (MIC) Assay***

Briefly. − 70 °C stored bacteria were sub-cultured two times overnight (O/N) at 37 °C on agar plates to obtain a pure single colony. 75 µL of desired concentration of metal salt stock (provided in the media) was added to 96 wells, 75 µL of 150-fold diluted 1.0 McFarland standardized inoculum of bacteria from colonies on LB media agar plate (equivalent 1.0 X 10^6^ CFU/ml) was then added to each well and the plate was incubated 24 hours at 37 °C in microplate shaker at 150 rpm (2). MIC was determined by reading the optical density at 600 nm (OD600), using a Thermomax microtiter plate reader with Softmax Pro data analysis software (Molecular Devices, Sunnyvale, CA). The last well which had no bacterial growth and OD absorption was defined as the MIC.

***Minimum Bactericidal Concentration (MBC) Assay and Recovery Potency of Bacteria.***

At the end of the MIC determination experiment, MBC was determined two different ways. I) 10 µL of each MIC well was transferred to 140 µL of the same fresh media (93% dilution of original antimicrobial concentration) in a new 96 plate and incubated for 24 hours at 37^◦^C in a microplate shaker at 150 rpm. MBC was determined by reading the optical density at 600 nm (OD600) of the recovery plates, using a Thermomax microtiter plate reader with Softmax Pro data analysis software (Molecular Devices, Sunnyvale, CA). The last well that had no bacterial growth by OD600 absorption was defined as the MBC. II) Using a multipin replica plater to transfer 2 µl to a LB agar plate, where the last dilution with no microcolony on the plate was the MBC. There was no difference observed between approaches.

***Synergism high-throughput susceptibility testing of microbial growth.***

“Checkerboard” arrangements of MBA combination were made in 96-well microtiter plates as previously described (3,4). When prepared, each checkerboard microtiter plate had one column negative control (just media without bacteria and MBAs), and one column growth controls as a positive control (without MBA, with media, and bacteria), 10 different concentrations of MBAs alone, 8 different concentrations of Ag alone, and each MBAs and Ag at 80 different combinations of concentrations were in each checkboard. For each checkboard analysis, the same MBIC and MBEC steps indicated above were conducted for surveying biofilm eradication, prevention, and synergism potency of MBAs combinations.

***Determination of FIC (fractional inhibitory concentration) for detection of synergism effects.***

The synergistic interactions rules suggested by the American Society for Microbiology for the testing of planktonic cells are used for both MIC and MBC synergism data obtained here(3). The fractional inhibitory concentration (FIC) and fractional eradication concentration (FEC) index for each combination of antimicrobial agents was calculated with the following formula:

FIC = MIC antibiotic A in combination/MIC antibiotic A alone + MIC antibiotic B in combination/MIC antibiotic B alone.

FEC = MBC antibiotic A in combination/MBC antibiotic A alone + MBC antibiotic B in combination/MBC antibiotic B alone.

To evaluate antimicrobial interactions, we used the lowest FIC/ FEC index method as described by Bonapace et al (5) and other studies (5-8). The lowest FIC/FEC obtained for all inhibitory or eradication combinations on the checkerboard was considered the FIC/FEC for the pair. Finally, FIC/FEC were interpreted as following: FIC/FEC <0.8= Synergy, FIC/FEC ≥0.8 and ≤1.2= Partial-synergy, FIC/FEC >1.2=Antagonistic (**Fig S1 and Table S1**).

***Reduced thiol (RSH) assay***

This assay is based on earlier work (9) The accuracy of the assay was assessed with a standard dilution of reduced glutathione (≥98%, Alfa Aesar, Germany) and oxidized glutathione (Sigma, USA). For preparing the standard, 1mM solution of each glutathione was serially diluted with 50 mM Tris/HCl pH 81:2 (150μL of 50 mM Tris/HCl pH 8 + 150μL of glutathione) for a total of 11 samples as a standard. Then 0.1 mM of Ellman's reagent 5,5’-dithiobis(2-nitrobenzoic acid) (DTNB) was added to each well. Samples were mixed and incubated for 30 minutes at 37 ºC in dark. Absorbances were measured at 412nm using a Thermomax microtiter plate reader with Softmax Pro data analysis software (Molecular Devices, Sunnyvale, CA).

For measuring the treated and untreated samples with metalloids. The bacteria were cultured in 3mL of SWF and were incubated at 37 ºC in a shaker incubator (150 rpm) to reach the OD600 of 0.08. Then treated with MIC concentrations of agents and untreated groups with PBS (as a negative control) and incubated at 37 ºC for 2 h in a shaker incubator (150 rpm). The bacterial cells were washed with PBS by centrifuging (10,000 rpm for 5 min) and discarding the supernatant. 1mL of 50 mM Tris/HCl pH 8.0, 5 mM EDTA, 0.1% SDS and 0.1 mM DTNB was added to each sample and vortexed. These cell suspensions were incubated at 37°C for 30 min, and then centrifuged in a microfuge for 1 min at 15000 g. Absorbances were measured at 412nm using a Thermomax microtiter plate reader with Softmax Pro data analysis software (Molecular Devices, Sunnyvale, CA).

***Iron Detection Ferene-S Assay***

The release of Fe^2+^ from the iron-sulfur cluster in *P. aeruginosa* was detected using a Ferene-S assay with the probe, 3-(2pyridyl)-5,6-bis(2-(5-furylsulfonic acid))-1,2,4-triazin (Sigma-Aldrich, St Louis, MO, USA) (10). The 10 mL of bacteria (OD600 of 0.08) were prepared in Tris-HCl buffer (20 mM, pH 7). The bacterial cells were washed with the same buffer by centrifuging (10,000 rpm for 5 min) and discarding the supernatant. The pellet (bacterial cells) was then lysed by sonication using a 250HT ultrasonic cleaner (VWR International), set at 60 Hz for 20 min in the same buffer. The samples were centrifuged (10,000 rpm for 5 min) and the supernatant was collected. The solution was treated with MIC concertation of metal(loid)s, negative control (dd H_2_O), positive control (90 ºC for 10 min), as well as ciprofloxacin and gentamycin as the antimicrobial comparator. Then, a 10 mM Ferene-S probe was added to each sample in a 96-well plate, and samples were incubated at 21 ºC in dark for 2 h. Absorbance was measured at 600 nm, using a Thermomax microtiter plate reader with Softmax Pro data analysis software (Molecular Devices, Sunnyvale, CA).

**Advantageous/harmful and oxidant/antioxidant survey in *Caenorhabditis elegans* as an animal model**

In total 4-6 synchronized L2 larvae were transferred on the plates containing each antibacterial agent. Four results were recorded for five days 1. Number of live worms, 2. Motility (head swing and body-bending frequency per minute), 3. Growth (body length and body width), 4. Regeneration (generation of offspring, size, number, and motility of them were considered) rates. For these experiments a high challenge load was chosen that was still not lethal. Here we used 10 times the MIC of the metals (silver nitrate (Ag) (1.25 mM) or potassium tellurite (Te) (2.5 mM), or the Ag-Te combination (1.25 mM Ag + 2.5 mM Te) as well as exposure to antibiotics gentamicin and ciprofloxacin (both at 12.5 µM). Thus, under this high antimicrobial load the food (*E. coli* bacterium cells) would die off within 24 hrs. We noted that these dead cells do not lyse and could continue to be food for the worms; however, towards the end of the experiment the worms would have run out of food. There was certainly no overcrowding on the plates. Yet still during this assay it is clear the worms grow, are motile and regenerate, and these parameters are different with different antimicrobial exposures.

Multiple fluorescent sensor probes were employed including dihydroethidium (DHE), 2′,7′-dichlorofluorescin diacetate (DCFH-DA), and naphthalene-2,3-dicarboxal-dehyde (NDA), all obtained from the Invitrogen (CA, USA) to detect the O_2·_^−^, ROS, and glutathione, respectively.

**Advantageous/disadvantage assessment assays of Ag and/or Te toward *Caenorhabditis elegans***

*C. elegans* handling, growth and manipulation were performed using standard methods (11). Initially, experiments were carried out using M9 liquid culture (12). However, because the working solution of different components had different densities, it affected the worm’s motility in the liquid media. Therefore, for the prevention of bias, all experiments were transferred to NGM plates (12).

The *C. elegans* wild-type strain N2 (Bristol) and *E. coli* OP50 at 10^9^ CFU/mL as a food source were used in this study (11,12). *E. coli* and 10 times the MIC of each antibacterial component were added to the agar plates. In total 4-6 synchronized L2 larvae in 10 µl of phosphate-buffered saline (PBS) were transferred on the plates, with three biological replicates for each condition (total n=12-18), and the exact number of larvae for each plate was recorded. Plates without any metalloids were used as controls. Treated and untreated groups were incubated at 20 °C in the dark and results were obtained for five days. Four results were recorded each day, 1. Number of live worms, 2. Motility (head swing and body-bending frequency per minute), 3. Growth (body length and body width), 4. Regeneration (generation of offspring, size, number, and motility of them were considered) rates. The treated and untreated groups were compared for reporting the ratio of the variables and results.

***Oxidant/Antioxidant* activity of the Ag and/or Te toward *Caenorhabditis elegans***

ROS levels lead to oxidative stress, inflammation, and cellular disruption, while reduced glutathione (GSH) is a cellular antioxidant against oxidative stress, and is essential to cellular protection (13). Multiple fluorescent sensor probes were employed including dihydroethidium (DHE), 2′,7′-dichlorofluorescin diacetate (DCFH-DA), and naphthalene-2,3-dicarboxal-dehyde (NDA), all obtained from Invitrogen (CA, USA) to detect the O_2·_^−^, ROS, and glutathione, respectively.

Different time frames and exposure times were examined to find the optimal conditions to see the differences between the test groups. After treatment of L2 nematodes with 10X MIC concentration of each agent in NGM plates at 20 °C, the O_2·_^−^and ROS levels were measured after 4 days, while glutathione levels were measured after 24h. The plates were washed, and the liquid was centrifuged (6000 rpm) 2 times with PBS. The nematodes were exposed to DHE (20 μM), DCF (10 μM), NDA (20 μM) probes to measure the O_2·_^−^, ROS, and glutathione, respectively. The nematodes were incubated with probes at 20 °C for 4 h. The liquid was gently washed 2 times with PBS buffer and the animals were examined on a fluorescence microscope (Zeiss axio imager Z1) with an identical exposure time (1.5 s). Densitometry and intensity analysis was performed by Fiji software (ImageJ).

***Supplementary results***

**Beneficial Vs toxicity of Ag and/or Te toward *Caenorhabditis elegans***

The Ag and Te exposed groups had almost the same advantageous/toxicity activity, while the Ag-Te exposure led to even more growth, motility, regeneration, and population numbers compared with the Ag, Te, and antibiotics groups; suggesting that the Ag-Te combination stimulates the health of the animal. Regeneration started after day four, so this variable is significant within our assessment.

**Oxidant/antioxidant activity of Ag and/or Te toward *Caenorhabditis elegans***

Free radical production is quite poisonous to the host; leads to cell wall lysis, biomolecular damage, disruption of cells, activation of the immune system, inflammation, and finally organ and tissue damage (14). For treated and untreated *C. elegans* groups, the ROS level was measured with the DCFH-DA probe. A noticeable difference was detected between metalloids when we exposed the animals individually and when we exposed them in a combination manner. More specifically, higher fluorescence intensity was observed in the Ag 22 (±3) RFU and Te 18.6 (±0.6) RFU in comparison with Ag-Te combination 12.5 (±1) RFU, as well as the control 11.5 (±1.8) RFU, ciprofloxacin 12.5 (±1.5) RFU, and gentamicin 11.3 (±1.6) RFU groups (**Fig S8**). Likewise, **Fig S9** shows the O_2·_^−^ level by using the DHE probe. The fluorescence intensity of Ag-Te combination group 9 (±1.6) RFU was quite close to control 10 (±2) RFU, ciprofloxacin 8 (±2.5) RFU, and gentamicin 9.8 (±2.8) RFU, but remarkably lower than Ag 17.3 (±1) RFU and Te 15 (±1.5) RFU groups.

Some interesting observations are seen for the effects of silver and tellurite on *C. elegans.* In Fig S8, we observe very brightly cyan stained oval shapes which are the embryos. It seems that tellurite leads to high ROS in embryos whereas there is little to no ROS (no cyan fluorescence) from the embryos upon silver alone exposure (Fig S8 panel d). These embryo’s also have ROS signal in the control but at much lower levels. Another curiosity is the brighter rod-shaped structures seen particularly in panel E (tellurite) but also to some extent panel A (control). We postulate that these may be hatched larvae. Normally, this animal lay eggs, which then hatch external to the parent.  However, if there is a lack of food, or if the egg laying muscles/structures are not functioning properly, then the female may retain the eggs, and they will hatch while still inside her. We expect that by end of experiment the worms had run out of food. This is referred to as a ‘bag of worms’ phenotype (15), but here it is not quite a ‘bag’. Regardless, tellurite exposure seems to be enhancing this phenotype.

***Supplementary Tables:***

**Table S1.** Bactericidal and Bacteriostatic concentrations for silver and tellurite and synergy scores against clinical isolates of *P. aeruginosa.*

| ***Clinical Samples ID*** | **Bactericidal (MBC) synergism** | | | | **Bacteriostatic (MIC) synergism** | | | |
| --- | --- | --- | --- | --- | --- | --- | --- | --- |
|  | ***FBC*** | ***Interpretation*** | ***Silver*** | ***Tellurite*** | ***FIC*** | ***Interpretation*** | ***Silver*** | ***Tellurite*** |
|  |  |  | ***Concentrations (mM)*** | |  |  | ***Concentrations (mM)*** | |
| Utah 3 | 0.18 | Synergy | Ag 0.312 | Te 0.125 | 0.19 | Synergy | Ag 0.156 | Te 0.031 |
| AR 091D06 | 0.56 | Synergy | Ag 0.039 | Te 0.25 | 0.29 | Synergy | Ag 0.039 | Te 0.016 |
| NP 10449 | 0.28 | Synergy | Ag 0.039 | Te 0.031 | 0.29 | Synergy | Ag 0.039 | Te 0.016 |
| IM 111008 | 0.157 | Synergy | Ag 0.039 | Te 0.025 | 0.28 | Synergy | Ag 0.039 | Te 0.031 |
| KS F6999 | 0.28 | Synergy | Ag 0.039 | Te 0.125 | 0.28 | Synergy | Ag 0.039 | Te 0.0625 |
| 14651 | 0.29 | Synergy | Ag 0.039 | Te 0.0625 | 0.31 | Synergy | Ag 0.078 | Te 0.0625 |
| 14672 | 0.28 | Synergy | Ag 0.156 | Te 0.0625 | 0.16 | Synergy | Ag 0.039 | Te 0.016 |
| 14690 | 0.52 | Synergy | Ag 0.039 | Te 0.0625 | 0.19 | Synergy | Ag 0.039 | Te 0.016 |
| 14717 | 0.186 | Synergy | Ag 0.078 | Te 0.0625 | 0.16 | Synergy | Ag 0.039 | Te 0.016 |
| Utah 4 | 0.28 | Synergy | Ag 0.156 | Te 0.125 | 0.19 | Synergy | Ag 0.156 | Te 0.016 |
| KR 080603 | 0.49 | Synergy | Ag 0.039 | Te 0.125 | 0.51 | Synergy | Ag 0.039 | Te 0.031 |
| SS 1705 | 0.62 | Synergy | Ag 0.039 | Te 0.125 | 0.16 | Synergy | Ag 0.039 | Te 0.016 |
| AS A7764 | 0.53 | Synergy | Ag 0.078 | Te 0.0625 | 0.29 | Synergy | Ag 0.039 | Te 0.016 |
| 7307 | 0.28 | Synergy | Ag 0.039 | Te 0.031 | 0.16 | Synergy | Ag 0.039 | Te 0.016 |
| 14655 | 0.56 | Synergy | Ag 0.078 | Te 0.0625 | 0.29 | Synergy | Ag 0.039 | Te 0.016 |
| 14673 | 0.31 | Synergy | Ag 0.078 | Te 0.125 | 0.19 | Synergy | Ag 0.039 | Te 0.016 |
| 14716 | 0.16 | Synergy | Ag 0.039 | Te 0.016 | 0.29 | Synergy | Ag 0.039 | Te 0.016 |
| PA01 | 0.69 | Synergy | Ag 0.312 | Te 0.125 | 0.53 | Synergy | Ag 0.039 | Te 0.0625 |

Concentrations shown are those of each metal giving the synergistic load

FIC/FBC <0.8=synergy; FIC/FBC ≥0.8 and ≤1.2=indifferent/additive; FIC/FBC >1.2=antagonistic.

Silver nitrate (Ag) with potassium tellurite (Te)

**Table S2.** Summary of cDNA samples for RNA-seq, *P. aeruginosa* treated with silver nitrate (Ag) with potassium tellurite (Te), and Ag-Te combination.

| **Sequenced sample** | **Number of reads (after trim)** | **Reads mapped (%)** | **Sample information** |
| --- | --- | --- | --- |
| Ag #1 | 35,633,067 | 99.09 | treated with 0.125 mM Ag |
| Ag #2 | 31,742,432 | 99.13 | treated with 0.125 mM Ag |
| Ag #3 | 26,994,460 | 99.09 | treated with 0.125 mM Ag |
| Te #1 | 28,666,175 | 99.28 | treated with 0.25 mM Te |
| Te #2 | 31,244,687 | 99.33 | treated with 0.25 mM Te |
| Te #3 | 38,064,451 | 98.92 | treated with 0.25 mM Te |
| Ag+Te #1 | 28,471,391 | 98.84 | treated with 0.125 mM Ag+0.25 mM Te |
| Ag+Te #2 | 23,577,868 | 99.15 | treated with 0.125 mM Ag+0.25 mM Te |
| Ag+Te #3 | 38,349,493 | 99.15 | treated with 0.125 mM Ag+0.25 mM Te |
| Control #1 | 30,069,284 | 99.64 | non treated (just bacteria + media) |
| Control #2 | 32,496,250 | 99.47 | non treated (just bacteria + media) |
| Control #3 | 28,035,266 | 99.31 | non treated (just bacteria + media) |

*Control group was treated with PBS

***Supplementary Figures:***


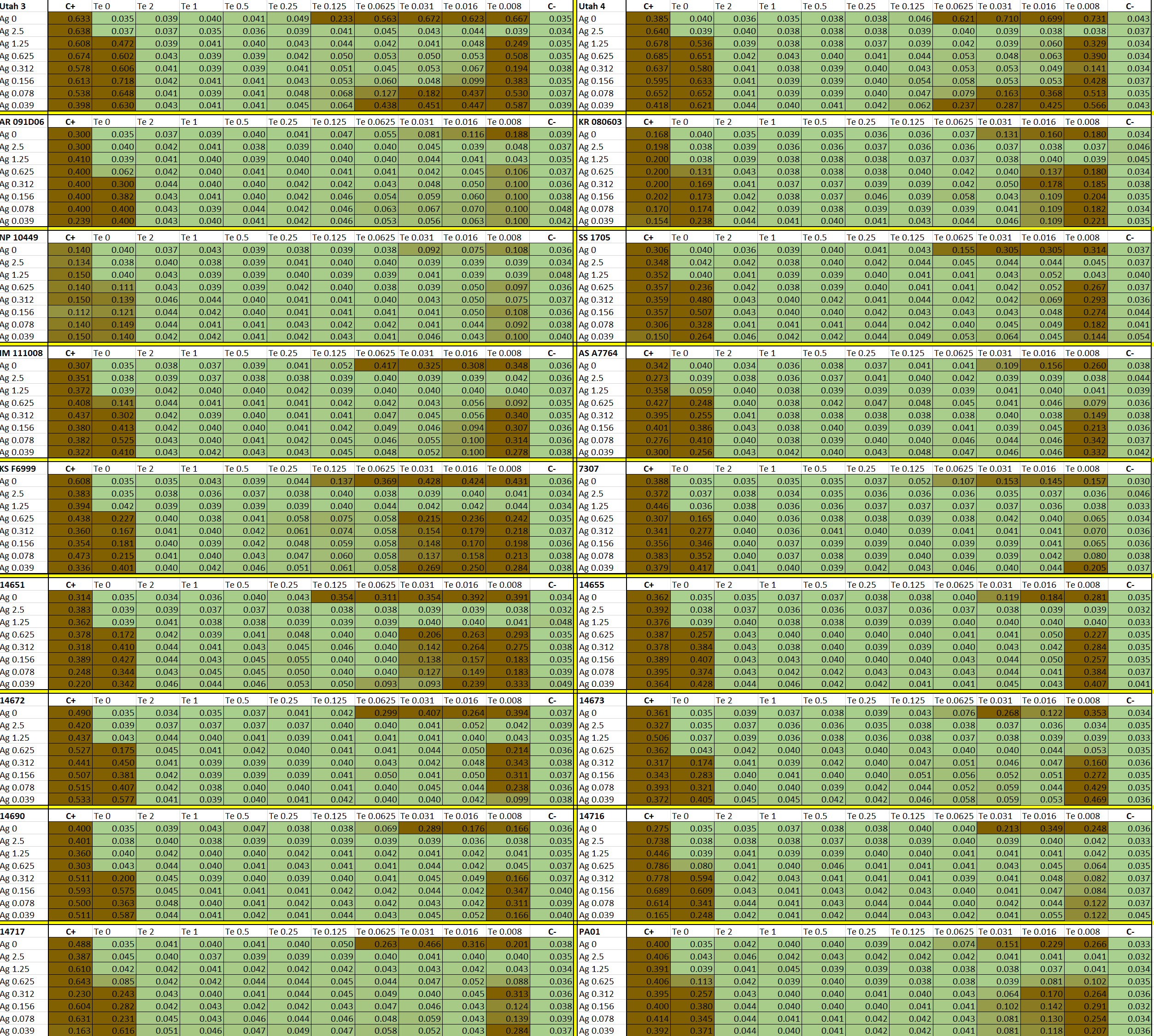


**Fig S1.** **Bacteriostatic (MIC) high-throughput susceptibility checkerboards of *P. aeruginosa* clinical isolates.** The clinical isolates treated, silver nitrate (Ag) with potassium tellurite (Te) individually and in combination, Ag is diluted from top to below and Te is diluted from left to right. The brown colour shows growth, and the green colour shows the wells that don’t have growth. Detailed synergism information is shown in **Table S1.**


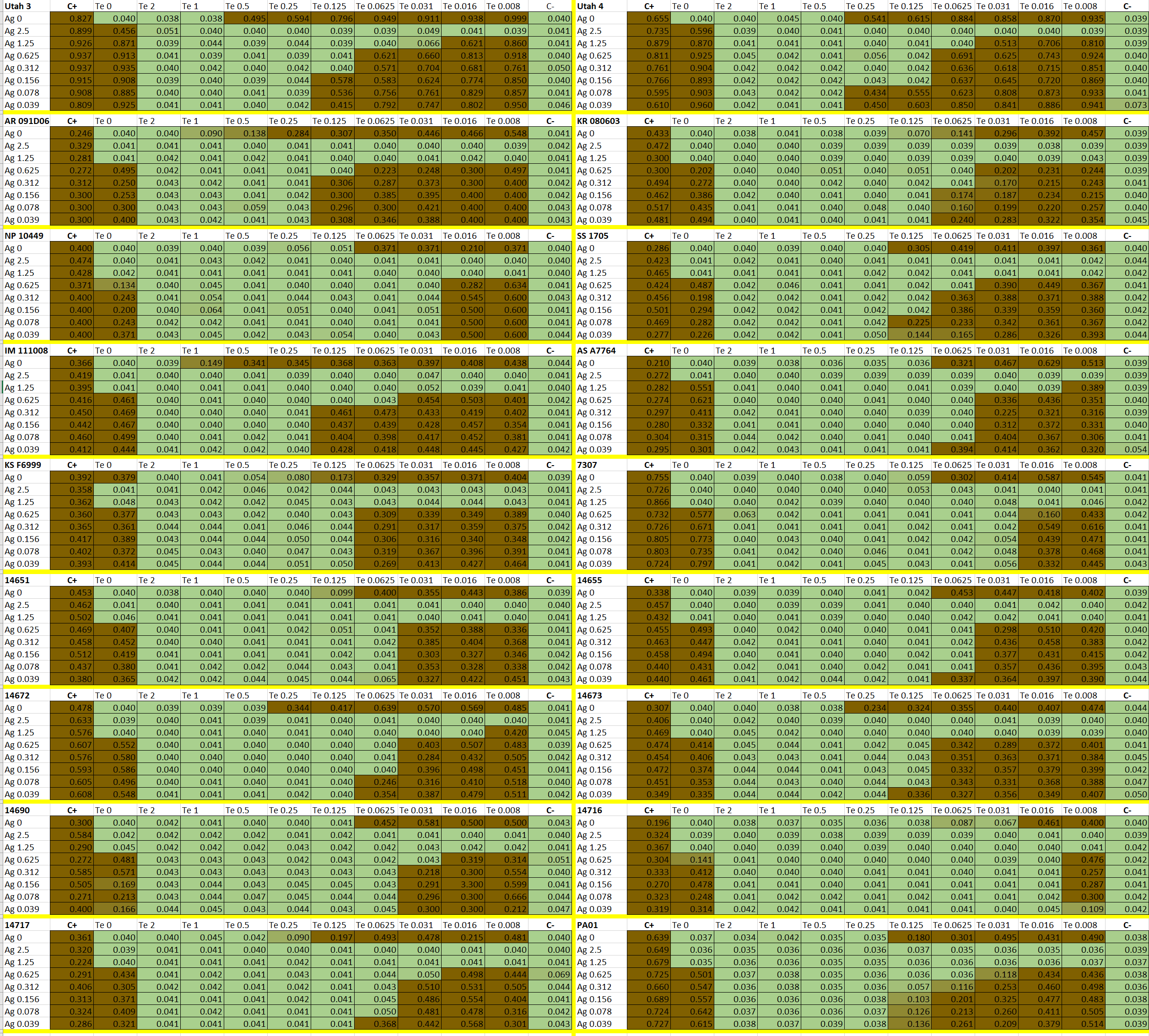


**Fig S2. Bactericidal (MBC) high-throughput susceptibility checkerboards of *P. aeruginosa* clinical isolates.** The clinical isolates were treated with silver nitrate (Ag), potassium tellurite (Te) individually and in combination, Ag is diluted from top to below and Te was diluted from left to right. The brown colour shows growth, and the green colour shows the wells without growth.

**
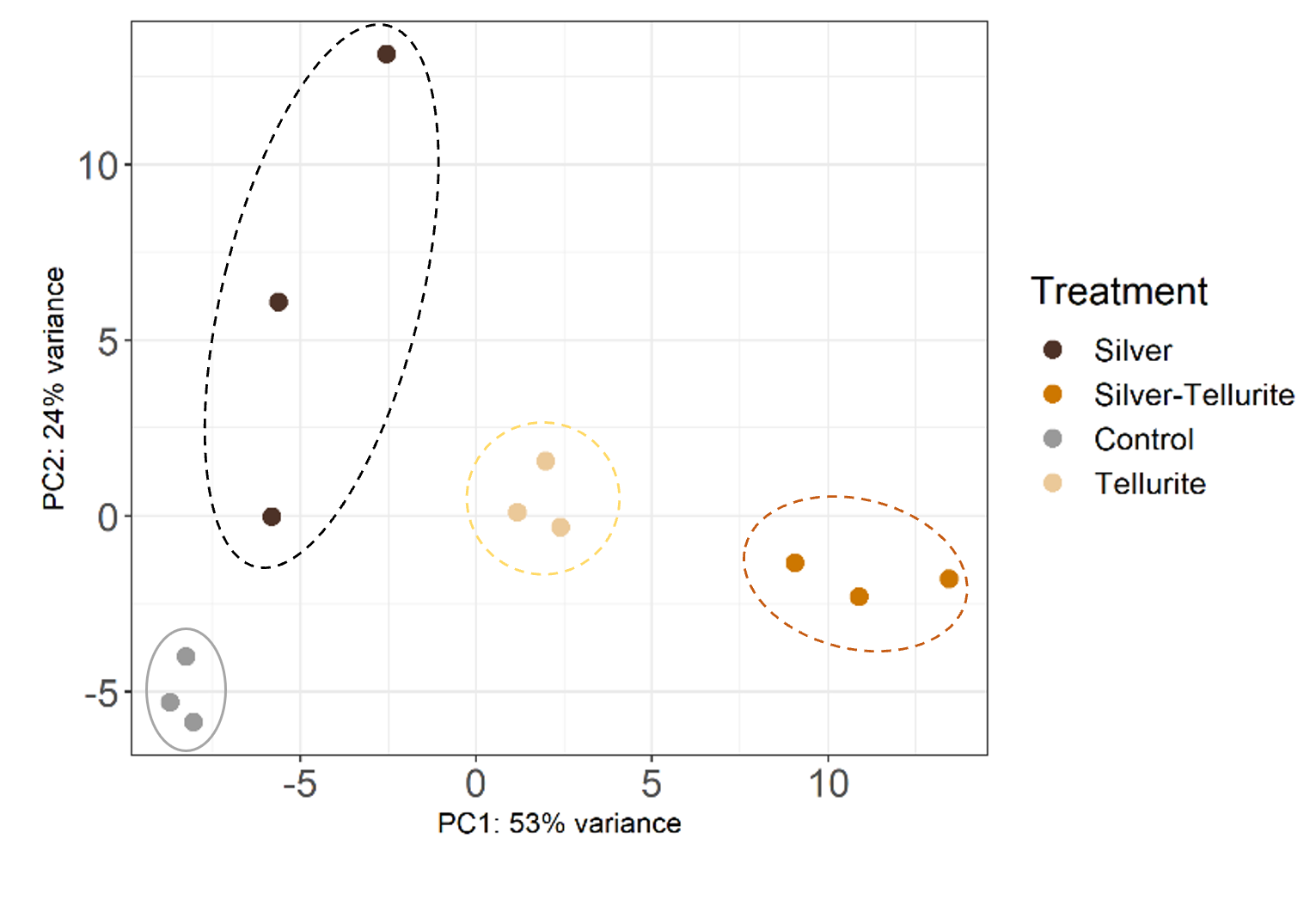
**

**Fig S3.** **Principal component analysis of RNA-seq data.** The PCA was performed using r-log transformed expression data.


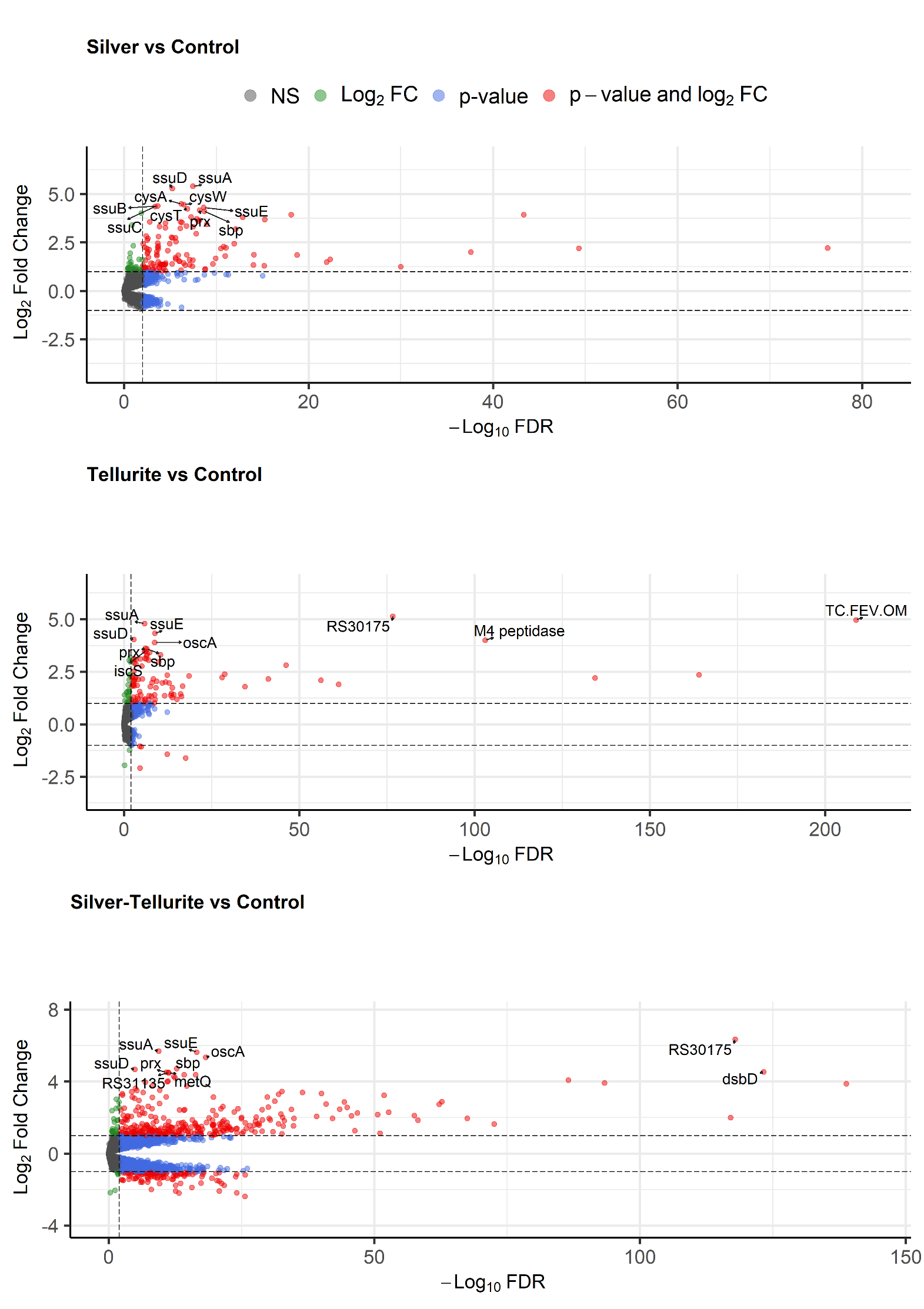


**Fig S4.** **Volcano plots of the distribution of gene expression for *P. aeruginosa* treated with silver nitrate (Ag), potassium tellurite (Te), and Ag-Te combinations**. Each circle represents a single gene. Differentially expressed genes are highlighted in red (FDR < 0.05 and |Log2 Fold Change| > 1). Labels indicate the top 10 differentially expressed genes with the highest positive Log2 Fold Change. Concentrations of the challenge are 0.125 mM silver nitrate (Ag) with 0.25 mM potassium tellurite (Te), and 0.125 mM Ag-0.25 mM Te combination.

**
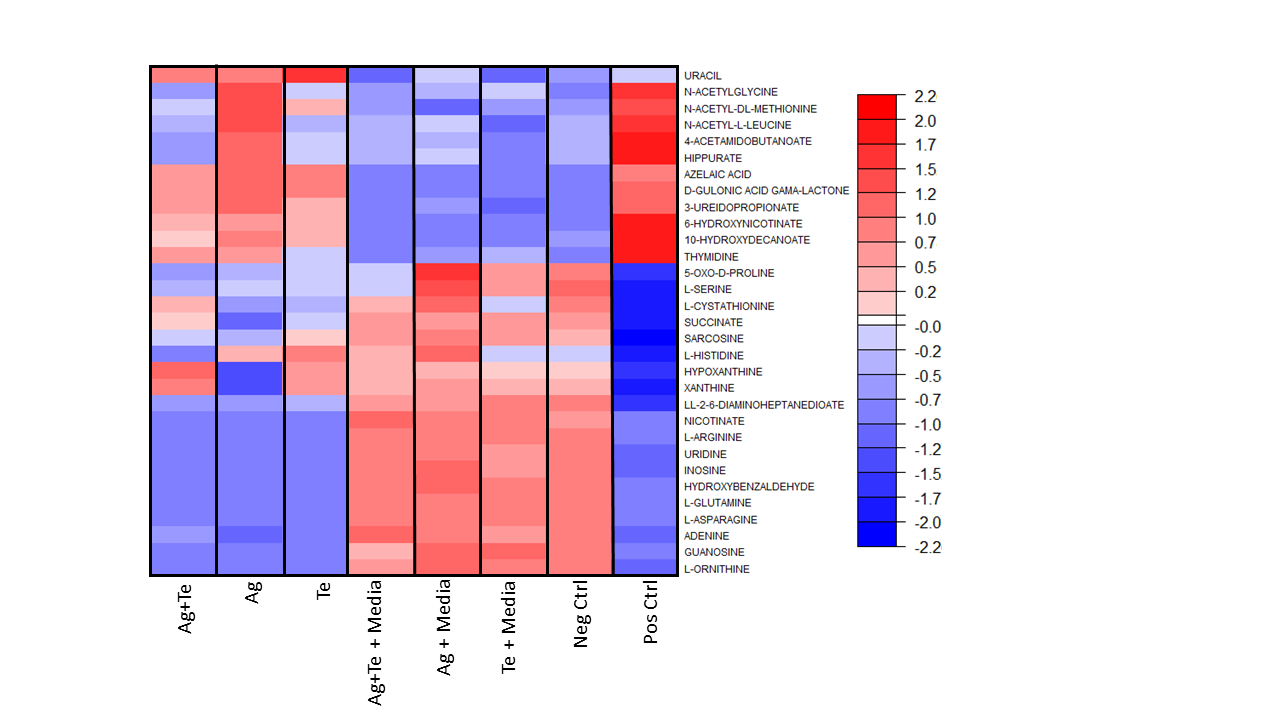
**

**Fig S5. Heatmap of *P. aeruginosa* extracellular metabolites.** Metabolomics experiments were performed under identical conditions as that of the transcriptomic experiment. Treatments with 0.125 mM silver nitrate (Ag), with 0.25 mM potassium tellurite (Te), or 0.125 mM Ag-0.25 mM Te combination in comparison with control (PosCtrl, treated with PBS) groups. Other controls of media alone (SWF) are also shown. The color for each treatment is an average of 3 replicates and the increase of metabolite is shown in dark red and the decrease of the metabolite is shown in blue color.

**
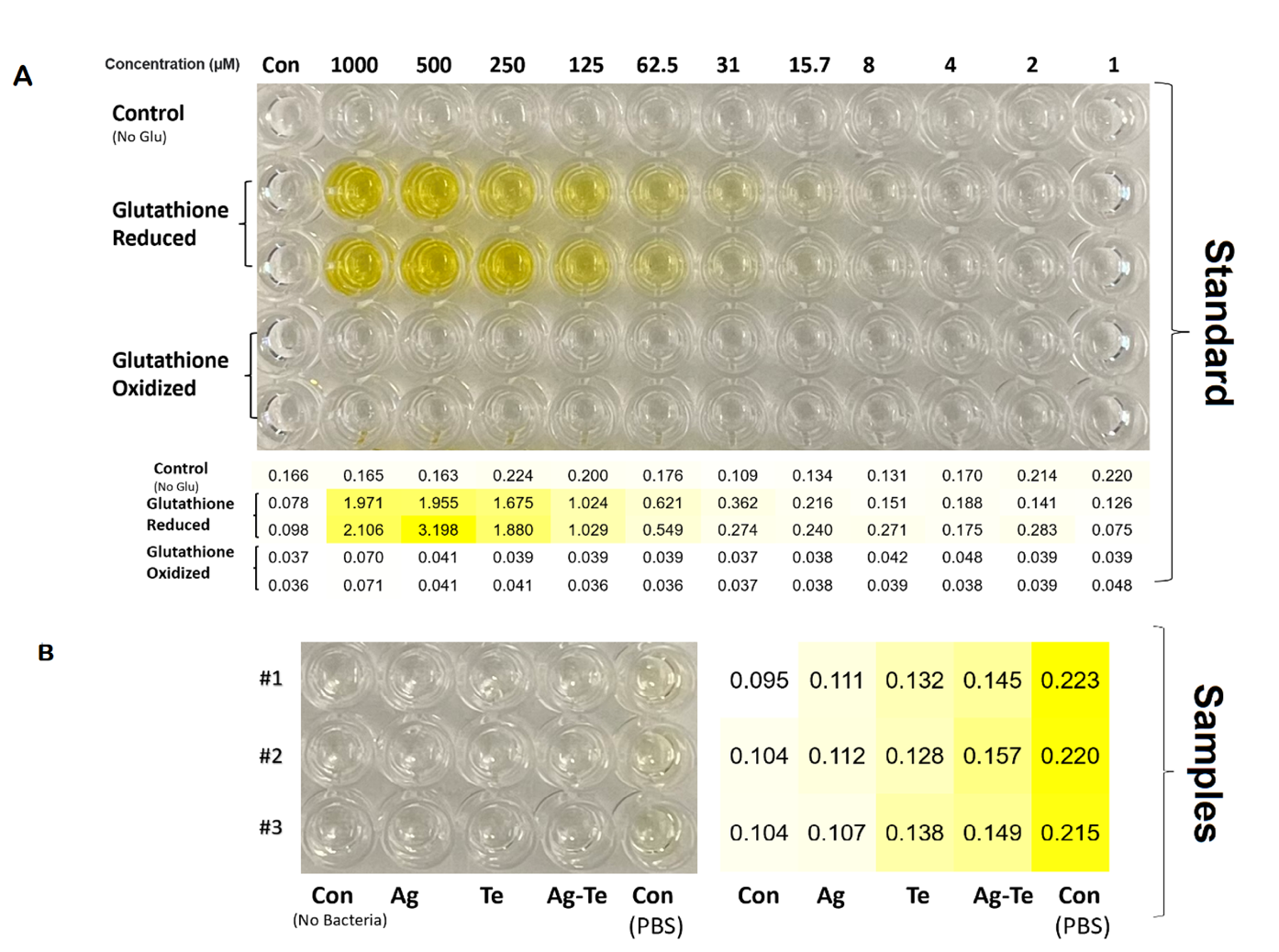
**

**Fig S6. Reduced thiol (RSH) level** **in the *P. aeruginosa* treated with silver nitrate (Ag) with potassium tellurite (Te), and Ag-Te combination** in comparison with control (Con), bacteria treated with PBS. **A.** Shows the standard, 1mM solution of glutathione reduced and glutathione oxidized serially diluted with Tris/HCl 1:2 for a total of 11 samples. The 50 mM Tris/HCl pH 8 and Ellman's reagent were used as the blank. **B.** Shows the RSH absorbance values in the samples with the naked eye (left) and plate reader (right), The Heatmaps (right panel) are added to visually illustrate the outcome, it shows the OD value with the microplate plate reader. Exposure concentrations of Ag, Te, Ag-Te (0.125 mM silver nitrate (Ag), with 0.25 mM potassium tellurite (Te), or 0.125 mM Ag-0.25 mM Te for the Ag+Te combination).


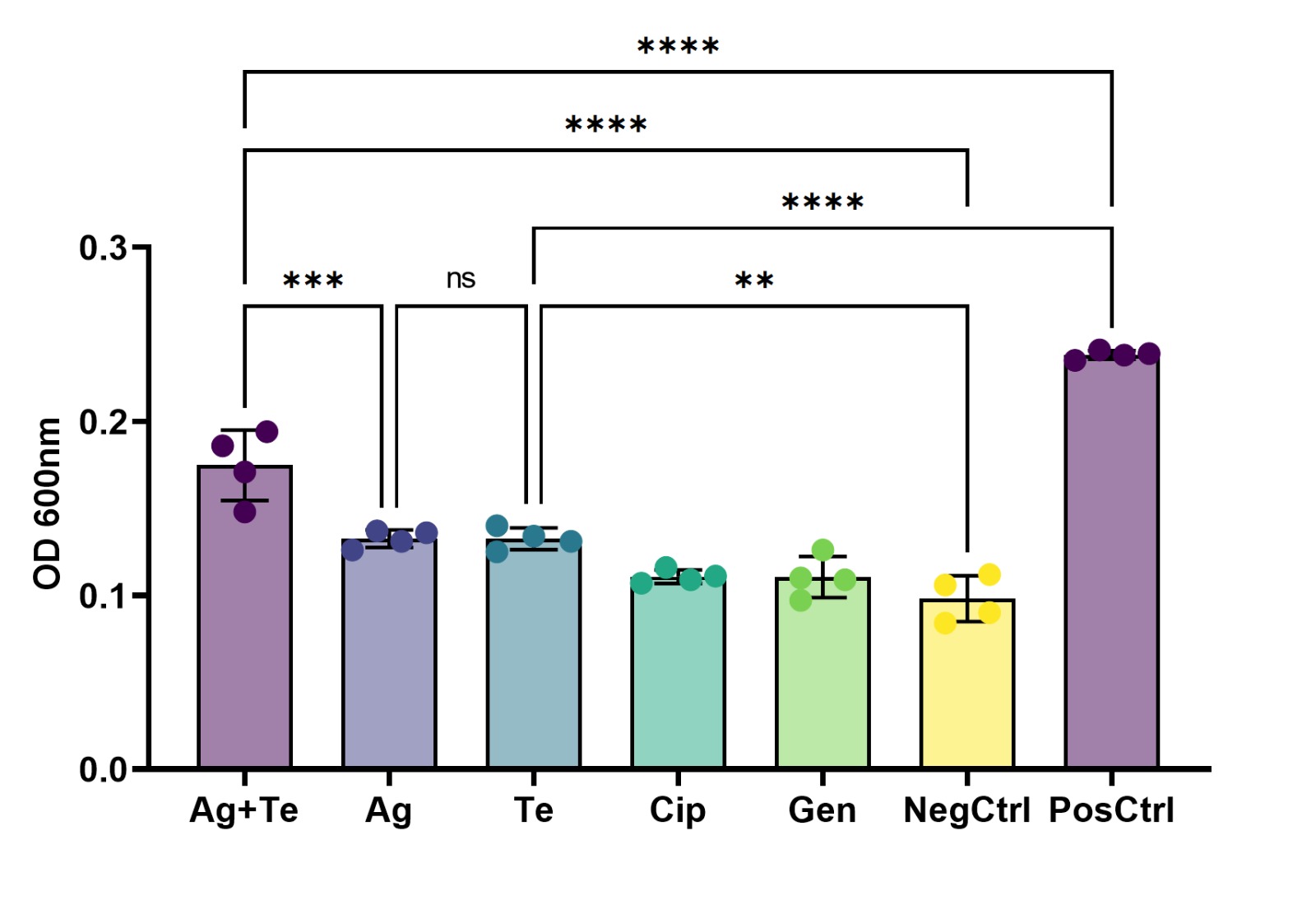


**Fig S7. Free iron [(ferrous) Fe^+2^] levels** **in a *P. aeruginosa* after exposure to antimicrobials.** Free iron [(ferrous) Fe^+2^] absorbance values in a *P. aeruginosa* lysate after 2 h treatment with MIC concentrations of Ag, Te, Ag-Te (0.125 mM silver nitrate (Ag), with 0.25 mM potassium tellurite (Te), or 0.125 mM Ag-0.25 mM Te for the Ag+Te combination). Ciprofloxacin and gentamycin used at 1.25 µM, negative control (NegCtrl, Bacteria+ working reagent), and bacterial boiled for 10 min to denature proteins and thus disrupt the [Fe-S] centres as a positive control (PosCtrl). The statistical differences between treatments were calculated by one-way ANOVA with post-hoc Tukey correction, where * p < 0.05; ** p < 0.01; *** p < 0.001; (n=4).

Cip: ciprofloxacin, Gen: gentamycin. Ag: silver nitrate, Te: potassium tellurite

**
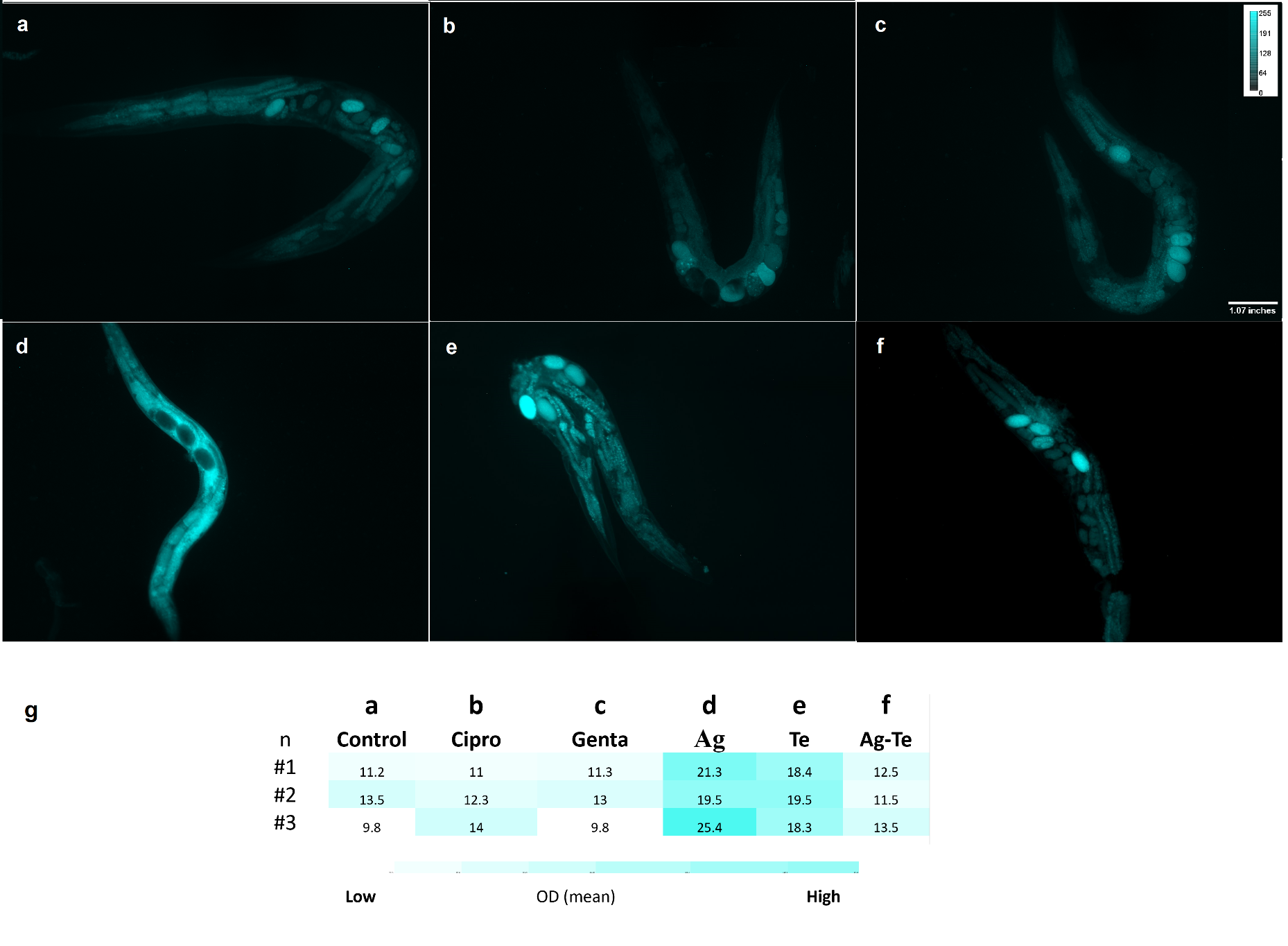
**

**Fig S8.** **Reactive oxygen species (ROS) levels** **in a *P. aeruginosa* after exposure to antimicrobials.** DCFH-DA staining was used to evaluate ROS levels. Fluorescence intensity from the DCFH-DA probe added to *C . elegans* was measured after exposure to control (PBS) (**a**), ciprofloxacin (12.5 µM) (**b**), Gentamicin (12.5 µM) (**c**), silver nitrate (Ag) at 1.25 mM (**d**), potassium tellurite (Te) at 2.5 mM (**e**), and Ag-Te combination (1.25mM + 1.25mM Te)(**f**). Heatmap on ROS levels in *C. elegans* for the treated and untreated groups showed in panel (**g**). n= trial number

**
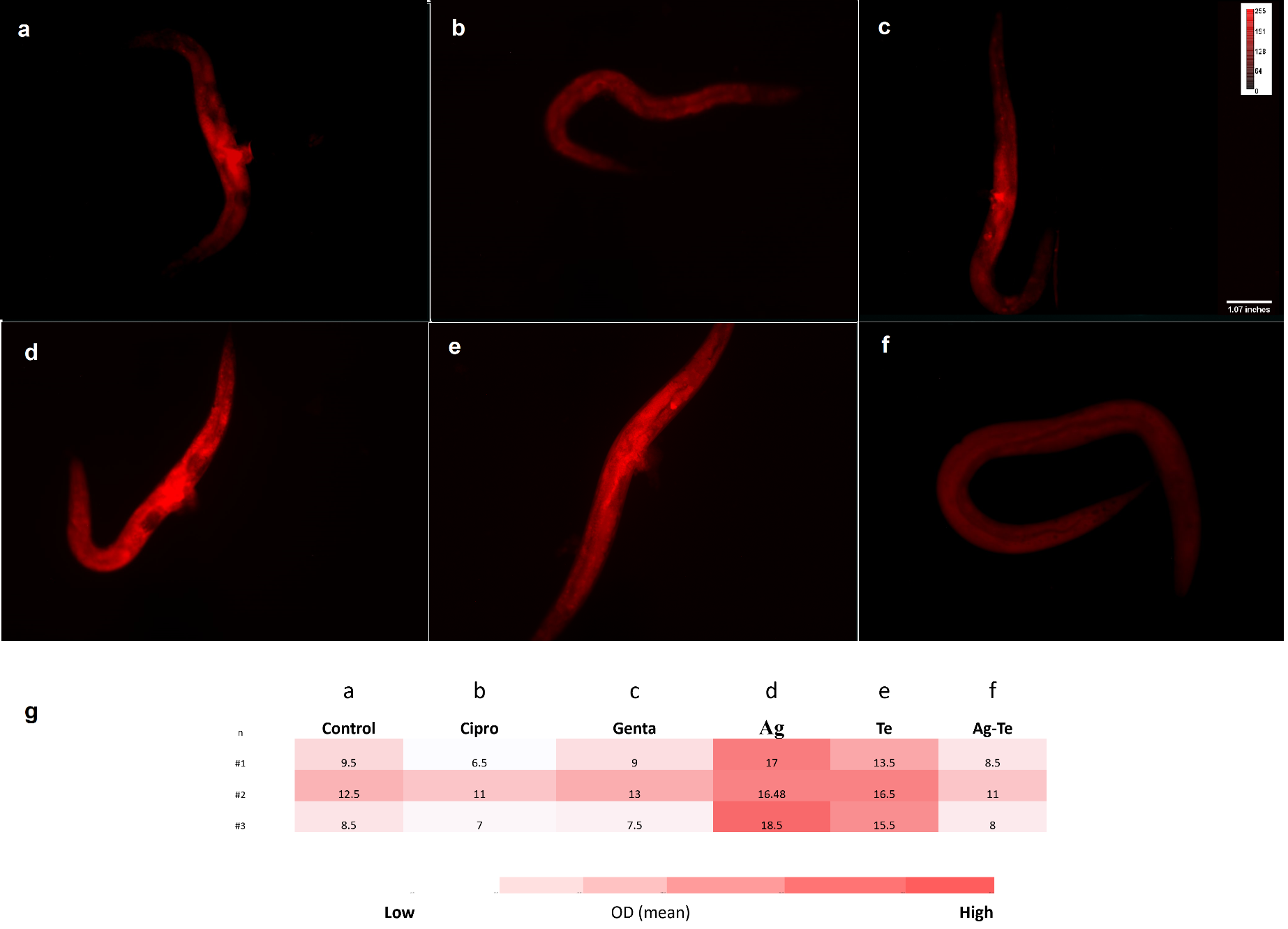
**

**Fig S9.** **O_2·_^−^ levels** **in a *P. aeruginosa* after exposure to antimicrobials.** DHE staining was used to evaluate the presense of O_2·_^−^. Fluorescence intensity of the DHE probe added to *C. elegans* was measured after exposure to control (PBS) (**a**), ciprofloxacin (12.5 µM) (**b**), Gentamicin (12.5 µM) (**c**), silver nitrate (Ag) at 1.25 mM (**d**), potassium tellurite (Te) at 2.5 mM (**e**), and Ag-Te combination (1.25mM + 1.25mM Te) (**f**). Heatmap on O_2·_^−^ levels in *C. elegans* for the treated and untreated groups shown in panel **(g)**. n= trial number


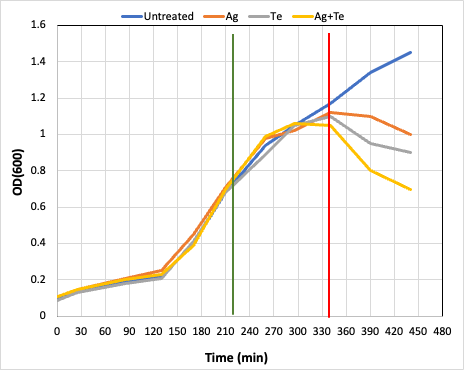


**Fig S10. Representative growth curve of Pseudomonas aeruginosa** ATCC 27853 cultured in simulated wound fluid. 3 mL culture was inoculated with 30 uL of overnight culture in a culture tube. The sample was challenged close to 0.8 OD_600_ at 0.125 mM silver nitrate (Ag; orange line), with 0.25 mM potassium tellurite (Te; grey line), or 0.125 mM Ag-0.25 mM Te (Ag+Te; yellow line) allowing comparison of growth to untreated (blue line). The red line represents the cell harvest time 2 hrs after challenge. The untreated culture became saturated overnight reaching an OD_600_ of 2.1, the challenged were found to be unviable.

***Supplementary References***

1. Lemire JA, Kalan L, Bradu A, Turner RJ. 2015. Silver oxynitrate, an unexplored silver compound with antimicrobial and antibiofilm activity. Antimicrobial agents and chemotherapy 59:4031-4039.
2. Monych NK, Turner RJ. 2020. Multiple Compounds Secreted by Pseudomonas aeruginosa Increase the Tolerance of Staphylococcus aureus to the Antimicrobial Metals Copper and Silver. mSystems 5.
3. Moody J. 2004. Synergism testing: broth microdilution checkerboard and broth macrodilution method. Clinical microbiology procedures handbook:1-28.
4. Harrison JJ, Turner RJ, Joo DA, Stan MA, Chan CS, Allan ND, Vrionis HA, Olson ME, Ceri H. 2008. Copper and quaternary ammonium cations exert synergistic bactericidal and antibiofilm activity against Pseudomonas aeruginosa. Antimicrobial Agents and Chemotherapy 52:2870-2881.
5. Bonapace CR, Bosso JA, Friedrich LV, White RL. 2002. Comparison of methods of interpretation of checkerboard synergy testing. Diagnostic microbiology and infectious disease 44:363-366.
6. Masuda G, Nakamura K, Yajima T, Saku K. 1980. Bacteriostatic and bactericidal activities of beta-lactam antibiotics enhanced by the addition of low concentrations of gentamicin. Antimicrobial agents and chemotherapy 17:334-336.
7. Den Hollander JG, Mouton JW, Verbrugh HA. 1998. Use of pharmacodynamic parameters to predict efficacy of combination therapy by using fractional inhibitory concentration kinetics. Antimicrobial agents and chemotherapy 42:744-748.
8. Richards R, Xing D. 1991. Evaluation of synergistic effects of combinations of antibacterials having relevance to treatment of burn wound infections. International journal of pharmaceutics 75:81-87.
9. Turner RJ, Weiner JH, Taylor DE. 1999. Tellurite-Mediated Thiol Oxidation in *Escherichia coli*. Microbiology, 145: 2549-2557.
10. Hennessy DJ, Reid GR, Smith FE, Thompson SL. 1984. Ferene—a new spectrophotometric reagent for iron. Canadian journal of chemistry 62:721-724.
11. Brenner S. 1974. The genetics of Caenorhabditis elegans. Genetics 77:71-94.
12. Stiernagle T. 2006. Maintenance of C. elegans (February 11, 2006), WormBook, ed. The C. elegans Research Community, WormBook, doi/10.1895/wormbook. 1.101. 1.
13. Boysen G. 2017. The Glutathione Conundrum: Stoichiometric Disconnect between Its Formation and Oxidative Stress. Chem Res Toxicol. 30(5):1113-1116.
14. Arulselvan P, Fard MT, Tan WS, Gothai S, Fakurazi S, Norhaizan ME, Kumar SS. 2016. Role of antioxidants and natural products in inflammation. Oxidative medicine and cellular longevity. 2016:5276130.
15. Mitchell DH, Stiles JW, Santelli J, Sanadi DR. 1979.Synchronous growth and aging of Caenorhabditis elegans in the presence of fluorodeoxyuridine. Journal of Gerontology. 34: 28–36.
