# Supplementary material for: Insights into the Synergistic Antibacterial Activity of Silver Nitrate with Potassium Tellurite against Pseudomonas aeruginosa
Source: Microbiol Spectr. 2023 Jul 6;11(4):e00628-23. doi: 10.1128/spectrum.00628-23 (PMC10433965; doi:10.1128/spectrum.00628-23)
Supplement: Supplemental file 3 — Supplemental material. Download spectrum.00628-23-s0003.docx, DOCX file, 0.01 MB [file spectrum.00628-23-s0003.docx]

**Supplementary Videos. Beneficial effects versus toxicity of the selected Metal(loids) in comparison with Antibiotics to *C. elegans*.** All treated groups with the silver nitrate (**Video S1**) with potassium tellurite (**Video S2**), and Ag-Te combination (**Video S3**) were compared with the gentamicin (**Video S 4**) exposed group. More detailed results were reported in Figure 8, treated groups were compared with untreated (control) group (**Video S 5**) as the ratio (%). (total n=15; 5 larvae × 3 biological repeats).   Ag: silver nitrate, Te: potassium tellurite. Additionally, arrows are pointing small animals that may not be clear in the videos.

**Video S1. Beneficial effects versus toxicity of the selected Metal(loids) in comparison with Antibiotics to *C. elegans*.** All treated groups with the silver nitrate. (total n=15; 5 larvae × 3 biological repeats).   Ag: silver nitrate.

**Video S2. Beneficial effects versus toxicity of the selected Metal(loids) in comparison with Antibiotics to *C. elegans*.** All treated groups with potassium tellurite. (total n=15; 5 larvae × 3 biological repeats).   Te: potassium tellurite

**Video S3. Beneficial effects versus toxicity of the selected Metal(loids) in comparison with Antibiotics to *C. elegans*.** All treated groups with Ag-Te combination. (total n=15; 5 larvae × 3 biological repeats).   Ag: silver nitrate, Te: potassium tellurite

**Video S4. Beneficial effects versus toxicity of the selected Metal(loids) in comparison with Antibiotics to *C. elegans*.** All treated groups with gentamicin. (total n=15; 5 larvae × 3 biological repeats).   Gen: gentamicin

**Video S5. Beneficial effects versus toxicity of the selected Metal(loids) in comparison with Antibiotics to *C. elegans*.** All treated groups with Phosphate buffered saline as a control. (total n=15; 5 larvae × 3 biological repeats).
